# Virtual Reality-Based Education for Patients Undergoing Radiation Therapy

**DOI:** 10.1007/s13187-020-01870-7

**Published:** 2020-09-24

**Authors:** Liam J. Wang, Brian Casto, Join Y. Luh, Samuel J. Wang

**Affiliations:** 1 Catlin Gabel High School, 8825 SW Barnes Rd, Portland, OR 97225 USA; 2 Department of Radiation Oncology, Salem Health, 875 Oak St SE, Salem, OR 97301 USA; 3 Department of Radiation Oncology, St. Joseph Hospital, 2700 Dolbeer St, Eureka, CA 95501 USA

**Keywords:** Virtual reality, Patient education, Cancer, Radiation oncology

## Abstract

We built a virtual reality (VR) application that runs on a commercially available standalone VR headset that allows patients to view a virtual simulation of themselves receiving radiotherapy. The purpose of this study was to determine if this experience can improve patient understanding of radiotherapy and/or reduce patient anxiety. We created software that reads data from our clinical treatment planning system and renders the plan on a life-size “virtual linear accelerator.” The patient’s CT simulation data is converted into a 3D translucent virtual human shown lying on the treatment table while visible yellow radiation beams are delivered to the target volumes in the patient. We conducted a prospective study to determine if showing patients their radiotherapy plan in VR improves patient education and/or reduces anxiety about treatment. A total of 43 patients were enrolled. The most common plans were 3D breast tangents and intensity-modulated radiotherapy prostate plans. Patients were administered pre- and post-experience questionnaires. Thirty-two patients (74%) indicated that they “strongly agree” that the VR session gave them a better understanding of how radiotherapy will be used to treat their cancer. Of the 21 patients who expressed any anxiety about radiotherapy beforehand, 12 (57%) said that the VR session helped decrease their anxiety about undergoing radiotherapy. In our single-institution, single-arm prospective patient study, we found that the majority of patients reported that the personalized VR experience was educational and can reduce anxiety. VR technology has potential to be a powerful adjunctive educational tool for cancer patients about to undergo radiotherapy.

**Electronic supplementary material:**

The online version of this article (10.1007/s13187-020-01870-7) contains supplementary material, which is available to authorized users.

## Introduction

Many cancer patients undergo radiotherapy as part of their treatment, a process which can be physically and psychologically demanding [1]. Cancer patients are often anxious about their treatment [2] or have misconceptions about the technology used in radiotherapy treatment. It is also common for patients to feel that they have a lack of control over their treatment. Currently, patients often do not have convenient access to general information about radiotherapy [3] or information about their personalized treatment plan. One method for alleviating these concerns is to educate patients about their ailment, the need for radiotherapy, and the relevant technology used during their daily radiotherapy sessions. A better understanding of the radiotherapy process could make treatment more tolerable and reduce patient anxiety.

Illustrating the objectives of radiotherapy and the process that takes place during treatment is difficult with existing visualization techniques [4]. The goal of radiotherapy is to utilize targeted ionizing radiation to selectively eliminate cancerous tissue while avoiding damage to adjacent normal tissues. When considering treatment options, the radiation oncologist must consider the location of a patient’s tumor in relation to other anatomy. It is challenging to describe these concepts to patients through conventional approaches because it is only possible to show a single static perspective of the treatment process in a picture or on a computer monitor. It is sometimes difficult for those who are unfamiliar with radiotherapy to understand how the two-dimensional visuals relate to their experience during therapy. Other researchers have created models that attempt to educate patients about their treatment by using two-dimensional pictures or educational videos [5]. With these conventional methods, it is often difficult for patients to visualize their own tumor size, shape, and location. Furthermore, it is difficult for them to understand the complex radiation therapy treatment process in which radiation beams enter the body from various angles and sometimes rotate in arcs around the patient. Others have used videos with 3D glasses [6] for patient education. These approaches use example tumors [7] and generic treatment plans to illustrate the treatment process [8] [9] [10], although they do not show the patient personalized information about their own tumor and treatment plan.

Virtual and augmented reality technology can be more effective at conveying information that requires a three-dimensional understanding of an environment. This novel VR technology is starting to be used in various fields in medicine [11], including medical research [12], surgical planning [13], medical training [14], patient therapy [15], and patient education [16]. It has also been shown that these immersive virtual experiences can promote improved recall [17].

This VR technology is well suited for multiple applications in the field of radiation oncology, since the process of designing, planning, and delivering radiotherapy requires a detailed knowledge of 3D spatial relationships between radiation beams and tumor size and location and normal human anatomy. Others have shown that VR has great potential for use by radiation oncologists, medical dosimetrists, and medical physicists, for designing and planning radiotherapy plans [18].

The purpose of this study was to determine if virtual reality technology can enhance education for cancer patients undergoing radiation therapy to improve their understanding of how radiotherapy will be used to treat their cancer. We sought to determine if a VR experience prior to starting radiotherapy could help them prepare in advance for what they will experience when receiving daily radiotherapy and potentially alleviate anxiety they may have regarding undergoing this treatment. We built an application that will allow patients to view an educational virtual reality (VR) experience showing the delivery of their radiotherapy treatment plan. With this system, a patient can get a preview of what it will be like before they begin their radiotherapy. The unique aspect of our approach is that the experience is personalized for each patient, allowing them to view a rendition of their own radiotherapy treatment in VR, and not just a generic treatment plan. We hypothesized that this VR educational tool will improve the patient’s understanding of their radiotherapy treatment, improve physician-patient communication, decrease their anxiety level about undergoing radiotherapy, and improve overall patient satisfaction with their cancer care.

## Methods

**Fig. 1:**
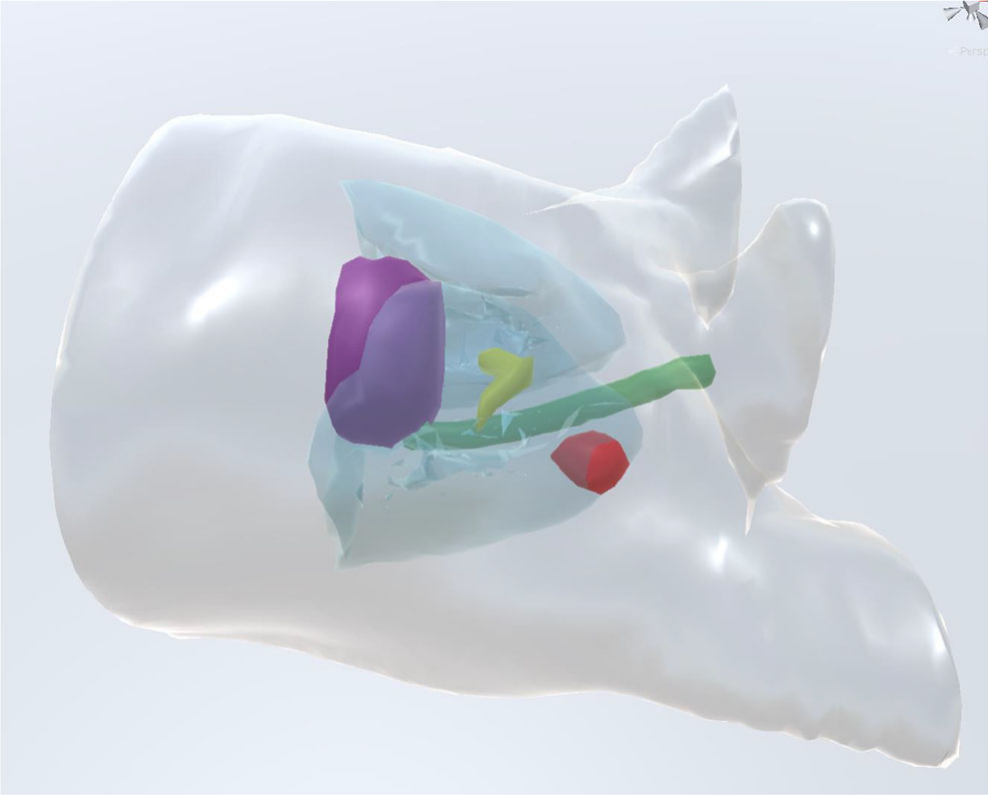
Example of anatomic structures taken from the radiotherapy treatment planning system, exported as DICOM, and converted into 3D geometry meshes as OBJ files for import into the virtual reality headset. Left upper lobe lung tumor planning target volume (red), lungs (light blue), heart (purple), spinal cord (green), carina (yellow).

**Fig. 2:**
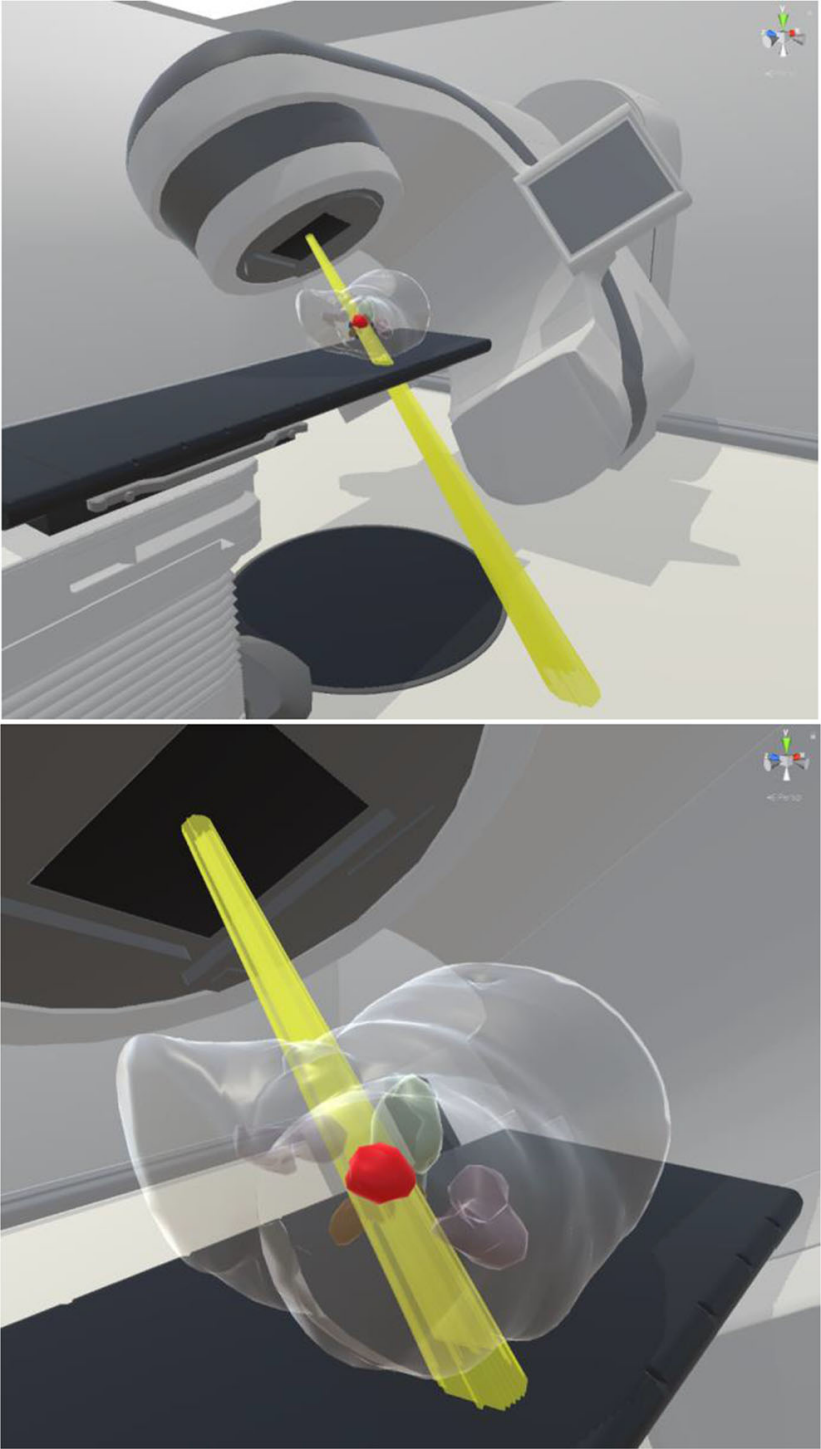
Virtual reality depiction of radiotherapy treatment delivery. (**a**) Overview of treatment vault, (**b**) example of a conformal radiation beam treating a prostate planning target volume (red)

We designed and built a novel virtual reality app that runs on the Oculus Quest [19], a commercially available standalone virtual reality headset. The patient’s radiotherapy plan is exported from our clinical treatment planning system in standard DICOM-RT [20] format. Before any further processing, all protected health information (PHI) is removed from the DICOM files, and then these data files are subsequently saved in a de-identified format with a context-free identifier assigned by the research staff. All DICOM-RT patient structures (e.g., target volumes, normal organs) are converted into 3D geometry meshes and saved in standard OBJ and MTL file formats, along with accompanying color and transparency information (Fig. 1). Radiotherapy beam information is extracted from the DICOM-RT file (jaw positions, MLC positions, gantry angle, collimator angle, table angle, monitor units) for all beam control points and saved into an intermediate file format, YAML [21], and then imported to the Oculus Quest. Using Unity [22], we created a virtual environment of a treatment vault that contains a 3D model of a linear accelerator with a fully movable patient table and gantry head (gantry and collimator angles) with adjustable multi-leaf collimator (MLC) leaves. The VR program displays a visual animation of the patient’s radiotherapy treatment plan played on the virtual linear accelerator (Fig. 2). Each beam in the treatment plan is played in sequence in real time based on the MU/min for each beam. Beams of radiation are shown as beams of yellow light. Gantry movement is modeled as smoothed arcs for dynamic conformal arcs or volumetric modulated arc-based therapy. MLC leaf motion is modeled for each control point, which dynamically changes the shape of the visible radiation beams in real time.

Since the radiotherapy experience varies widely for each patient depending upon the type of radiotherapy plan, this program shows the patient the specific treatment they will be receiving to allow them to get a more realistic advance preview of what will be happening in the vault before their first day of treatment. When the patient dons the headset, they will feel as if they are actually in a radiotherapy treatment vault with a life-sized linear accelerator. Because the headset is untethered and uses room-scale VR, the user can walk around and view the radiotherapy treatment delivery from any perspective in the room. A full-scale 3D rendering of the relevant part of the patient’s body is shown in position on the treatment table with a translucent body contour so that the target volume and internal normal organs can be seen. This allows the patient to see how the radiation beams are shaped and targeted specifically to the size, shape, and location of their tumor(s), conforming to the target volume of interest while avoiding adjacent normal structures in their body.

### Study Design

We conducted a single-arm, single-institution prospective clinical trial to determine if showing patients a VR rendition of their RT treatment plan would improve understanding of their radiotherapy plan, improve physician-patient communication, or decrease anxiety about radiotherapy. This study was approved by our hospital’s Institutional Review Board.

Patients were recruited for participation via a research flyer from a single radiation oncology clinic. Patients over age 18 planning to receive radiotherapy were eligible candidates for this study. Patients were excluded if they had vision or hearing impairment or if they had a known history of vertigo, motion sickness, or vergence-accommodation conflict associated with 3D media headsets.

Study patients participated in one research session which lasted about 30 min. The research session was usually scheduled about 1 to 2 days prior to the patient’s radiotherapy treatment start date. The patient’s treatment plan was loaded into the VR headset. A separate 2D monitor that mirrored the headset view was available in the room for family members and research staff to view what the patient was seeing in real time. The patient’s treating radiation oncologist was present during the viewing to narrate and explain details to the patient in real time during the experience. Since the Oculus Quest is a standalone room-scale VR headset, ambulatory patients were encouraged to walk around the room to view the scene from different angles, and they could get up close to the treatment table to see details inside their translucent body while the yellow radiotherapy beams were being delivered. Patients with balance or mobility issues that were deemed a fall risk were asked to remain seated in a chair, and the research staff helped move the chair around so the patient could view the virtual scene from different vantage points around the room.

### Evaluation

We designed a questionnaire to ascertain each patient’s current knowledge about their cancer, their understanding of how radiotherapy treatment works, and their anxiety level regarding the prospect of undergoing radiotherapy (Table 2). We used a standard numerical 5-point Likert Scale for all answers, 1 = strongly disagree, 2 = disagree, 3 = neutral, 4 = agree, and 5 = strongly agree. Participants completed the same questionnaire both before and after the VR experience, and both a paired *t* test and the Wilcoxon Rank Sum test were used to test statistical significance of differences for each question.

## Results

From September 2019 through March 2020, a total of 43 participants completed this study. Table 1 shows the basic demographics of the enrollees. (An additional 6 participants had been enrolled but were unable to complete the study because the research program was temporarily paused due to the COVID-19 coronavirus restrictions at our hospital.)

**Table 1:** Patient demographics

	*n*	**%**
Age (mean)	67.3	
Female	22	(51)
Plan type		
3D	22	(51)
IMRT	21	(49)
Disease site		
Breast	19	(44)
Prostate	12	(28)
Lung	4	(9)
Esophagus	3	(7)
Rectal	3	(7)
Endometrial	2	(5)
	43	(100)

Table 2 shows the statistical results of the pre- and post-survey differences. Both the paired *t* test and the Wilcoxon Rank Sum test showed statistically significant differences between the pre- and post-questionnaires for all questions.

**Table 2:** Questionnaire to ascertain each patient’s current knowledge about their cancer, their understanding of how radiotherapy treatment works, and their anxiety level regarding the prospect of undergoing radiotherapy and statistical results of the pre- and post-survey differences

		Before	After	Paired *t* test
	Question	Mean (sd)	Mean (sd)	*p* value
1	I understand where the cancer is located in my body	4.65 (0.686)	4.88 (0.324)	0.011
2	I understand the size of my cancer	4.09 (0.97)	4.67 (0.52)	< 0.001
3	I understand how radiation beams will be aimed to treat my cancer	3.95 (0.90)	4.91 (0.29)	< 0.001
4	I understand why radiation beams may give me side effects	4.14 (0.77)	4.58 (0.63)	< 0.001
5	I understand what I will feel like when I am laying on the treatment table each day	3.60 (1.05)	4.65 (0.57)	< 0.001
6	I am anxious about getting radiation treatment	3.19 (1.24)	2.63 (1.21)	< 0.001
7	I am anxious about my cancer	3.37 (1.07)	2.98 (1.20)	0.005
Additional Questions				
A1	I now have a better understanding of how radiation will be used to treat my cancer		4.73 (0.60)	
A2	The headset and 3D virtual reality program was easy to use		4.81 (0.39)	
A3	Viewing the virtual reality program made me feel uncomfortable, have headaches, or nausea		1.40 (0.76)	

A total of 40 participants (93%) indicated that they “agree” or “strongly agree” that the VR session gave them a better understanding of how radiotherapy will be used to treat their cancer. Of the 21 patients who expressed any anxiety about radiotherapy beforehand, 12 (57%) said the VR session helped decrease their anxiety about undergoing radiotherapy.

After the VR session, 41 participants (95%) stated “agree” or “strongly agree” that they had a good understanding of how they would feel when lying on the treatment table, compared with only 22 (51%) before the session.

The number of participants indicating that they understood why radiation might cause them side effects increased from 33 (77%) to 40 (93%) after the VR session.

After the VR educational session, more participants expressed an understanding of the size (42) and location (43) of their cancer compared with before the session (29 and 40, respectively).

A diverging stacked bar graph comparing the pre- and post-survey results for each question is available in the online Appendix.

## Discussion

There are several unique advantages of our approach.

Unlike watching a traditional flat screen wearing 3D glasses [7–9, 23–26], the advantage of using a standalone VR headset is that it enables a more immersive 3D experience giving the patient the sensation that they are actually in the treatment vault. It allows the patient to walk around the virtual linac room and get up close to their virtual translucent body on the table to see the treatment from different angles.

A unique feature of our approach is that we showed each patient their own actual radiotherapy plan, not a generic plan. This enables patients to personally identify with what they are seeing in the virtual treatment room. It allows them to see how the radiation beams are tailored to exactly conform to the size and shape of their own cancer and minimize exposure to their adjacent normal organs. We found that patients became more fully engaged when they realized they were actually seeing a life-sized virtual rendition of themselves on the treatment table and could see how the radiation plan was customized for them. Many patients commented on the size and location of their tumors when seeing this for the first time. We wrote custom software that takes a standard DICOM-RT export from our clinical treatment planning system and converts it into a format compatible with the VR headset. Our software automates the process of conversion and import so we were able to load the VR headset with patient-specific radiotherapy plans very quickly, allowing us to easily conduct multiple educational sessions in 1 day.

Watching a virtual rendition of the radiotherapy process often triggered patients to think of additional questions to ask the staff. We found that the clinician narrator played an important role in explaining to the patient what they were seeing, such as how much dose is actually being received to adjacent organs at risk. Often, the clinician encouraged the patient to walk to a different vantage point to see from a different perspective or to approach closer to see small details such as the separation between beam edge and the heart, or the small amount of anterior rectal wall included in an IMRT beam arc. We found that the VR sessions improved communication by providing another valuable opportunity for additional dialog between physician and patient.

Many platforms for virtual and augmented reality are currently commercially available, including Oculus Quest, Oculus Rift, Oculus Go, HTC Vive, and Windows Mixed Reality. For our research study, we selected the Oculus Quest because it is a standalone system, easily portable to any room, and uses room-scale VR, which allows the patient to walk around the virtual vault to see the ongoing treatment from any vantage point. Our system can be easily ported to other VR devices that use the Unity platform, such as the Oculus Rift or the HTC Vive.

This study has several limitations.

This was a small study that enrolled a limited number of patients from a single radiation oncology clinic.

In the design of our study, we elected to have only a single intervention group and did not enroll a separate control group since this was a preliminary proof-of-concept study. Also, rather than restricting enrollment to just one cancer type, we decided to allow all disease sites in order to test out feasibility with a wide variety of radiotherapy plans. Consequently, it may be more difficult to draw generalizable conclusions from our results given the disparate cancer types studied. However, in our ad hoc subset analyses, there was no significant difference in understanding improvement or anxiety decrease when comparing by disease site (breast, prostate, other) or radiotherapy plan type (3D, IMRT).

We only administered the post-intervention survey at one time point—immediately following the VR experience. Another option would be to survey patients repeatedly at multiple time points during and after their radiotherapy course to determine if this VR experience results in a more durable improvement over time compared with traditional patient education methods.

### Future Work

At the start of the COVID-19 pandemic, our research project was temporarily paused as our hospital worked on establishing new guidelines for patient and visitor restrictions and sanitizing standards. In the next phase of this research project, the VR headsets will be cleaned and sanitized between patients using the new standards established at our hospital for cleaning hospital equipment for the COVID-19 era. In addition to standard face masks required for all patients entering our facility, patients will also wear single-use disposable VR face covers before putting on the VR headset. We use an untethered type of VR headset that uses a room-scale tracking design so that these educational sessions can be conducted in a large room allowing research staff and patients to remain 6 ft apart at all times.

In our post-intervention survey, 30 (70%) participants stated that they would be very interested in having a downloadable version of this experience on their mobile device so that they could take it home and watch it again or show family and friends. We have created an augmented reality (AR) version that runs on iOS mobile platforms and have plans to introduce this alternate platform in our next clinical patient trial. This augmented reality version will allow the user to walk around the room with the mobile device and see a “window” into the life-sized 3D virtual radiotherapy treatment room and see the radiation being administered to their virtual body on the table. The advantage of this approach is that it does not require the use of a specialized VR headset and can be viewed on the patient’s own mobile device such as an iPhone or an iPad, and patients would be able to download and keep a copy on their own mobile device to view again later or to show family and friends.

In the future, we plan to conduct a larger study and include multiple institutions to assess the generalizability of this personalized VR approach to patient education. We also plan to measure whether this VR educational experience improves patient compliance during treatment [27] and potentially improve outcomes.

## Conclusions

In conclusion, we have designed and built an application to render a 3D simulation of a patient’s clinical radiotherapy treatment plan in a commercially available standalone virtual reality headset. This tool can be used to augment patient education for those about to start radiotherapy treatment. Our preliminary clinical patient trial demonstrates that this VR experience gives many patients a better understanding of how radiotherapy will be used to treat their cancer, and it can decrease their anxiety about undergoing radiotherapy treatment.

## Electronic Supplementary Material

ESM 1 (DOCX 220 kb)

## Data Availability

All data and materials and custom software code comply with all field standards.
