# Supplementary material for: Virtual Reality-Based Education for Patients Undergoing Radiation Therapy
Source: J Cancer Educ. 2020 Sep 24;37(3):694–700. doi: 10.1007/s13187-020-01870-7 (PMC7512212; doi:10.1007/s13187-020-01870-7)
Supplement: Supplementary file 1 — (DOCX 220 kb) [file 13187_2020_1870_MOESM1_ESM.docx]

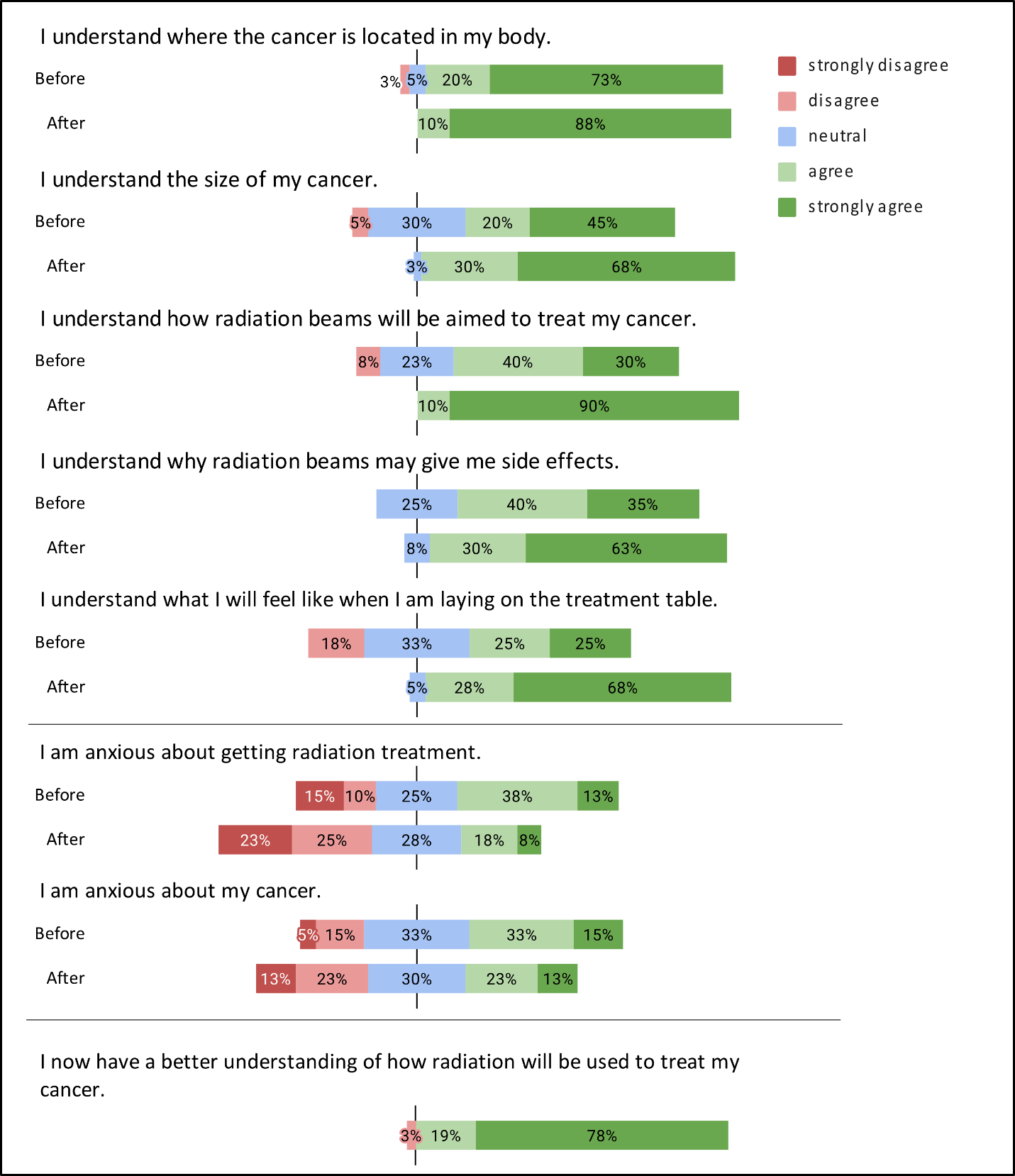


**Appendix:** Diverging stacked bar graph comparing the pre- and post- survey results for each question
